# Maternal age, perimenopause, and Alzheimer's disease on maternal cognitive and behavioral health

**DOI:** 10.1002/alz70855_101073

**Published:** 2025-12-23

**Authors:** Alesia V Prakapenka

**Affiliations:** ^1^ Midwestern University, Downers Grove, IL, USA

## Abstract

**Background:**

Pregnancy and perimenopause are female‐specific reproductive events that are accompanied by drastic shifts to circulating ovarian hormone milieu. Ovarian hormones modulate female physiology, biology, and behaviors, and may, at least in part, contribute to increased Alzheimer's disease (AD) risk in females compared to males. We utilized translational mouse models to investigate the relationship between age, pregnancy, and perimenopause on cognitive and behavioral health in healthy and AD females.

**Methods:**

Wildtype (WT; C57BL/6J) and 3xTg‐AD mice were purchased from Jackson laboratories. Virgin females were paired with WT males for first pregnancy and birth to occur at 2‐m‐o, 5‐6‐m‐o, or 9‐m‐o. A subset of virgin females was not paired with a male to serve as virgin controls. Postnatal maternal behaviors were assessed on the maternal pup retrieval task and with home cage maternal care evaluations. At 10‐m‐o, females received intraperitoneal 160 mg/kg/day VCD for 10 days to model perimenopause. Perimenopausal maternal behaviors were evaluated on the water radial arm maze for spatial working memory, open field task for locomotor activity, elevated plus maze for anxiety‐like behaviors, and forced swim task for depressive‐like behaviors.

**Results:**

Number of successful first pregnancies and size of the first litter decreased as maternal age increased. Maternal pup retrieval time was similar between the 2‐m‐o WT and AD dams. When compared to the 2‐m‐o WT dams, age at first birth did not impact maternal pup retrieval time for the 5‐6‐m‐o WT dams. However, the 5‐6‐m‐o AD dams took longer to retrieve pups to the nest than the 2‐m‐o AD dams and the 5‐6‐m‐o WT dams. Of note, at the end of the task, all 5‐6‐m‐o AD dams had two or more pups outside of the nest. When maternal care behaviors were scored in the home cage environment, WT and AD dams exhibited similar behaviors across the 2‐hr period.

**Conclusions:**

AD genotype and maternal age at first birth modulate maternal behaviors during the postnatal period, an effect that may be influenced by behavioral changes associated with AD progression. Additional analyses address the relationship between postnatal and perimenopausal maternal behaviors in the context of healthy and AD female aging trajectories.

